# Levodopa is associated with reduced development of new-onset geographic atrophy in patients with age-related macular degeneration

**DOI:** 10.1186/s40662-024-00412-2

**Published:** 2024-11-06

**Authors:** Kyle S. Chan, Nitika Aggarwal, Shannon Lawson, Nick Boucher, Mathew W. MacCumber, Jeremy A. Lavine

**Affiliations:** 1 Department of Ophthalmology, Feinberg School of Medicine, Northwestern University, Chicago, IL USA; 2 Vestrum Health, Naperville, USA; 3 Department of Ophthalmology, Rush University Medical Center, Chicago, IL USA; 4 Illinois Retina Associates, Chicago, IL USA

**Keywords:** Age-related macular degeneration, Geographic atrophy, L-DOPA, Levodopa

## Abstract

**Background:**

Geographic atrophy (GA) is a significant cause of vision loss in patients with age-related macular degeneration (AMD). Current treatments are limited to anti-complement drugs, which have limited efficacy to delay progression with significant risk of complications. Levodopa (L-DOPA) is a byproduct of melanin synthesis that is associated with reduced development of neovascular AMD. In this study, we determined if L-DOPA was associated with a reduced likelihood of new-onset GA.

**Methods:**

We performed a retrospective analysis in the Vestrum Health Retina Database. We included eyes with non-neovascular AMD without GA and 1–5 years of follow-up. Eyes were divided into two groups. Exposed to L-DOPA before or on the date of non-neovascular AMD without GA diagnosis, and eyes not exposed to L-DOPA. We extracted age, sex, AREDS2 status, dry AMD stage, smoking history, and conversion rate to GA at years 1 through 5. Propensity score matching was used to match L-DOPA and control groups. Cox proportional hazard regression, adjusting for age, sex, AMD severity, AREDS2 use, smoking status, and L-DOPA use was employed to calculate hazard ratios for new-onset GA detection.

**Results:**

We identified 112,089 control and 844 L-DOPA exposed eyes with non-neovascular AMD without GA. After propensity score matching, 2532 control and 844 L-DOPA exposed eyes remained that were well-matched for age, sex, AMD severity, AREDS2 use, and smoking status. We found that L-DOPA exposure was associated with a significantly reduced likelihood (HR = 0.68, 95% CI: 0.48–0.95, *P* = 0.025) of new-onset GA detection.

**Conclusion:**

L-DOPA use was associated with reduced detection of new-onset GA.

## Background

Non-neovascular age-related macular degeneration (AMD) can progress to geographic atrophy (GA), leading to vision loss in an estimated one million individuals in the United States [1]. GA involves loss of the photoreceptor and retinal pigment epithelium (RPE) layers, leading to significant visual impairment [2]. Treatment options for GA have traditionally been limited. Evidence suggest that Age-Related Eye Disease Study (AREDS) supplements do not have a significant benefit on the development or progression of GA [3, 4]. Recently, complement pathway inhibitors, such as pegcetacoplan or avacincaptad pegol, have shown promise in reducing GA area enlargement [5, 6]. However, long-term data is lacking, visual benefits have not been found, and significant complications can occur [7]. Therefore, there is an unmet need for therapies to prevent vision loss from GA.

Patients of the Caucasian race demonstrate an increased risk for both non-neovascular and neovascular AMD [8]. Although race is multi-factorial, macular choroidal melanin levels are increased in African American compared to Caucasian patients [9]. Levodopa (L-DOPA), a drug commonly used for the treatment of Parkinson’s disease, is a byproduct of the melanin biosynthesis pathway and can cross the blood-ocular barrier [8]. Model studies have shown that L-DOPA binds to the G-protein coupled receptor GPR143 expressed on RPE [10]. GPR143 agonism leads to increased expression of anti-angiogenic pigment epithelial derived factor, decreased expression of pro-angiogenic vascular endothelial growth factor, and decreased exosome production [11, 12]. Retrospective large database studies have shown that L-DOPA exposure is associated with delayed age of onset of both AMD and neovascular AMD, decreased intravitreal anti-vascular endothelial growth factor (anti-VEGF) injection burden, and reduced conversion to neovascular AMD [13–15].

While L-DOPA exposure is associated with reduced onset of neovascular AMD, it is unclear whether similar associations exist for new-onset GA detection. Since L-DOPA can dilate the choroidal vasculature and reduce oxidative stress [16–18], we hypothesized that L-DOPA exposure reduces the likelihood of GA development. To test this hypothesis, we retrospectively identified patients with non-neovascular AMD without GA from the Vestrum Health Retina Database. Patients were stratified into L-DOPA exposure and no L-DOPA exposure groups. Propensity score matching (PSM) was used to match age, sex, AMD severity, Age-Related Eye Disease Study 2 (AREDS2) vitamin use, smoking status, and number of years of follow up between groups. Survival analysis was performed utilizing Cox proportional hazard regression. We found that eyes exposed to L-DOPA were 32% less likely to demonstrate new-onset GA.

## Methods

We performed a retrospective analysis of patients in the Vestrum Health Retina Database between January 2014 and July 2023. The Vestrum Health Retina Database is a large deidentified electronic health records database encompassing 1.5 million unique patients and 11 million encounters with United States retina specialists. The Rush University Medical Center Institutional Review Board/Ethics Committee ruled that approval was not required for this study (ID 5403). Consent was not required in this retrospective database study in which all data was deidentified. This study adhered to the tenets of the Declaration of Helsinki. Inclusion criteria included eyes in the database with a diagnosis of non-neovascular AMD without GA [by international classification of disease (ICD) diagnosis codes] and at least 1 year of follow-up. The cohort was divided into two groups: (1) eyes that had L-DOPA exposure and (2) eyes with no history of L-DOPA exposure. Exclusion criteria were any eyes with exposure to L-DOPA occurring after the time of non-neovascular AMD diagnosis or when a variable of interest was unknown including smoking status, use of AREDS2 vitamins, and AMD severity. Detection of new-onset GA was defined by ICD diagnosis code use in the follow up period. Fundus photography, optical coherence tomography, and fundus autofluorescence are unfortunately unavailable for confirmatory review in the Vestrum deidentified database.

We extracted patient age, sex, smoking status, AMD severity, and AREDS2 vitamin use. To address potential confounding and other sources of bias inherent in observational studies, we employed PSM to the nearest neighbor with 3:1 ratio with replacement. We used the 'MatchIt' package in R to perform nearest neighbor matching between L-DOPA exposure and control groups. The covariates included in the propensity score model were age, sex, severity of AMD (early vs. intermediate), AREDS2 use, smoking status, and number of years of follow-up. The validity of the matching was assessed by comparing the standardized difference of covariates between the L-DOPA exposed and control groups before and after matching. Balance of covariates was considered satisfactory if there were no significant differences in distributions after matching (Table 1). The unmatched dataset comprised 154,559 observations, whereas the matched dataset consisted of 3376 observations (Table 1).

**Table 1:** Unmatched and matched cohorts

Parameter	Unmatched			Matched		
	No L-DOPA	L-DOPA	Std. Mean Diff	No L-DOPA	L-DOPA	Std. Mean Diff
	(n = 112,089)	(n = 844)		(n = 2,532)	(n = 844)	
Age （years）						
Mean	75.5	78.3	0.4481	78.4	78.3	− 0.0036
Standard deviation	9.2	7.2		7.2	7.2	
Sex						
Male	42,573 (38%)	425 (50%)	0.2475	1,273 (50%)	425 (50%)	0.0016
Female	69,516 (62%)	419 (50%)	− 0.2475	1,259 (50%)	419 (50%)	− 0.0016
Severity						
Early	43,332 (39%)	288 (34%)	− 0.0957	854 (34%)	288 (34%)	0.0083
Intermediate	68,757 (61%)	556 (66%)	0.0957	1,678 (66%)	556 (66%)	− 0.0083
AREDS use						
No	46,120 (41%)	345 (41%)	− 0.0055	1,036 (41%)	345 (41%)	− 0.0008
Yes	65,969 (59%)	499 (59%)	0.0055	1,496 (59%)	499 (59%)	0.008
Smoking status						
Never	69,805 (62%)	557 (66%)	0.0785	1,673 (66%)	557 (66%)	− 0.0017
Active	7,339 (6.5%)	20 (2.4%)	− 0.2747	56 (2.2%)	20 (2.4%)	0.0104
Former	34,945 (31%)	267 (32%)	0.0099	803 (32%)	267 (32%)	− 0.0017
Maximum follow up (years)						
1	1,353 (1.2%)	14 (1.7%)	− 0.1875	31 (1.2%)	14 (1.7%)	− 0.0158
2	39,977 (36%)	334 (40%)		1,002 (40%)	334 (40%)	
3	26,221 (23%)	242 (29%)		725 (29%)	242 (29%)	
4	18,375 (16%)	113 (13%)		340 (13%)	113 (13%)	
5	26,163 (23%)	141 (17%)		434 (17%)	141 (17%)	
Mean	2.8	2.5	0.1886	2.6	2.5	0.0326
Standard deviation	1.3	1.2		1.3	1.2	
Developed GA						
Year 1	1,353 (1.2%)	14 (1.7%)		33 (1.3%)	14 (1.7%)	
Year 2	1,873 (1.7%)	12 (1.4%)		61 (2.4%)	12 (1.4%)	
Year 3	1,479 (1.3%)	6 (0.7%)		38 (1.5%)	6 (0.7%)	
Year 4	1,150 (1.0%)	6 (0.7%)		32 (1.3%)	6 (0.7%)	
Year 5	834 (0.7%)	2 (0.2%)		18 (0.7%)	2 (0.2%)	
Total (1–5 years)	6,689 (6.0%)	40 (4.7)		182 (7.2%)	40 (4.7%)	
Years from incident diagnosis to censor date* [mean (standard deviation)]						
Developed GA	2.2 (1.3)	1.7 (1.1)		2.1 (1.2)	1.7 (1.1)	
Did not develop GA	2.9 (1.5)	2.6 (1.3)		2.6 (1.4)	2.6 (1.3)	

^*^Censor date is the earliest date of GA development (for developed GA eyes) or latest recorded visit for eyes that did not develop GA

*Std. Mean* *Diff*. = standard mean difference; *L-DOPA =* levodopa; *AREDS* = Age-Related Eye Disease Study; *GA* = geographic atrophy

Survival analysis was completed to estimate the association between L-DOPA use and the time to new-onset GA among the propensity score matched cohort. We created a mixed effects Cox regression model to calculate hazard ratios (HRs) and the corresponding 95% confidence intervals (CIs), adjusting for age, sex, AMD severity, AREDS2 use, smoking status, and L-DOPA use as fixed effects while accounting for correlation between different eyes of the same patient as random effects.

## Results

We performed a retrospective analysis of eyes with a diagnosis of non-neovascular AMD to determine if L-DOPA exposure was independently associated with conversion to GA in the 1–5 year follow-up period. Our initial cohort included 153,494 control eyes and 1065 L-DOPA exposed eyes. After excluding eyes with unknown smoking history, unreported AREDS use, and indeterminant AMD stage, our sample size was 112,089 (73% of total) control eyes and 844 (79% of total) L-DOPA exposed eyes. The L-DOPA exposed group was older, included more male patients, had more advanced AMD severity, and less active smokers. After PSM, the matched group included 2523 control and 844 L-DOPA exposed eyes. The groups were well-matched in terms of age, sex, AMD severity, AREDS2 use, smoking status, and years of follow-up (Table 1). We found that 4.7% of L-DOPA exposed eyes developed new-onset GA compared to 7.2% of control eyes (Table 1). Time to GA development was 1.7 years in L-DOPA exposed eyes compared to 2.1 years in control eyes (Table 1).

**Fig. 1:**
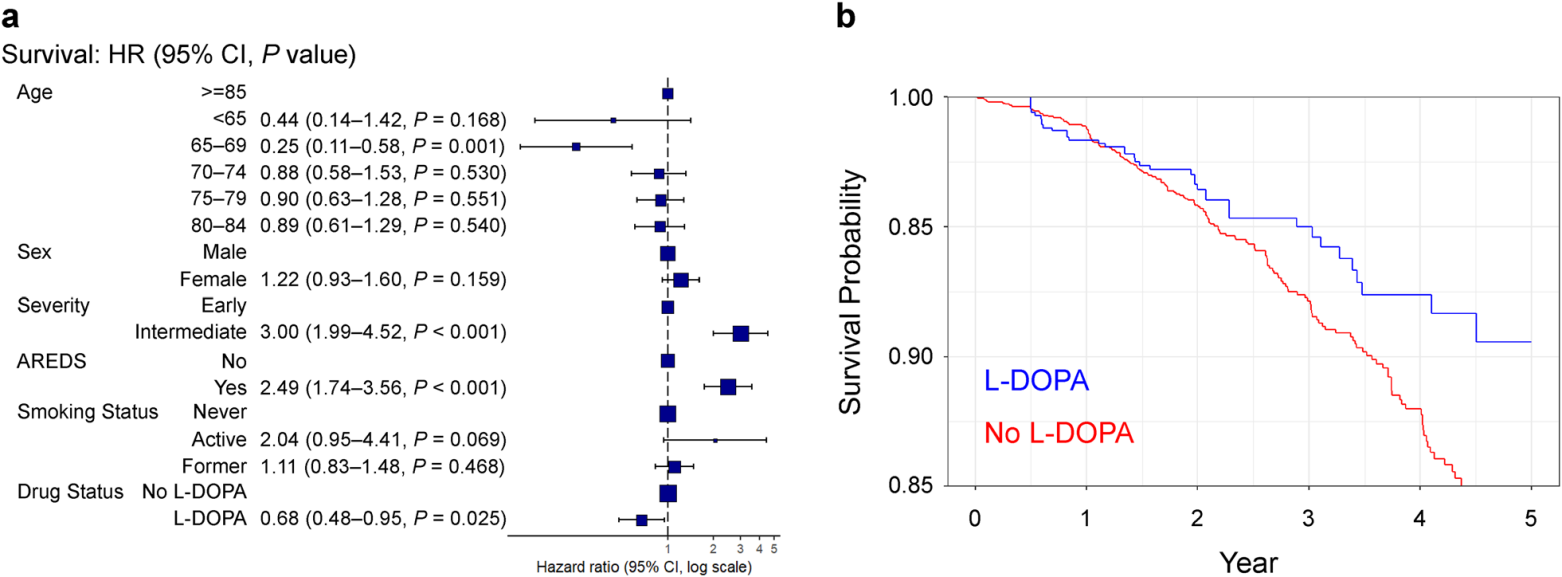
L-DOPA is associated with reduced likelihood of incident GA. **a** Forrest plot of hazard ratios and 95% confidence intervals (CIs) for the effect of age, sex, AMD severity, AREDS2 use, smoking status, and L-DOPA exposure on new-onset GA. **b** Kaplan–Meier survival curve for probability of conversion to new-onset GA. L-DOPA, levodopa; GA, geographic atrophy; AMD, age-related macular degeneration; AREDS2, Age-Related Eye Disease Study 2

Survival analysis for progression to new-onset GA was performed using a Cox proportional hazard regression model. We found that L-DOPA exposure was associated with a significantly reduced likelihood (HR = 0.68, 95% CI: 0.48–0.95, *P* = 0.025) of incident GA (Fig. 1a–b). In addition, younger age (age 65–69 years, HR = 0.25, 95% CI: 0.11–0.58, *P* = 0.001) was also associated with reduced likelihood of new-onset GA detection (Fig. 1a). No significant associations were found for sex or smoking status. Finally, both intermediate AMD severity (HR = 3.00, 95% CI: 1.99–4.52, *P* < 0.001) and AREDS2 vitamin use (HR = 2.49, 95% CI: 1.74–3.76, *P* < 0.001) were associated with a greater likelihood of incident GA (Fig. 1a).

## Discussion

We found that L-DOPA exposure was associated with a 32% risk reduction in new-onset GA detection over a period of 1–5 years. These results support the hypothesis that L-DOPA exposure may reduce the likelihood of GA development. Since complement pathway inhibitors, such as pegcetacoplan or avacincaptad pegol, only reduce GA growth by 15%–20% [5, 6] and have the potential for devastating complications like occlusive retinal vasculitis [7], alternative treatments to prevent GA onset are important for the future of dry AMD treatment.

There are several potential mechanisms that may explain the effects of L-DOPA exposure on the development of GA. The pathophysiology of GA is multifactorial and includes oxidative stress at the RPE, complement-induced inflammation, exosomes, and choriocapillaris loss [2, 19–21]. Exogenous L-DOPA dilates the choroidal vasculature via the D1/D5 dopamine receptor in animal studies [17]. In agreement, patients with Parkinson’s disease taking L-DOPA demonstrate significantly greater choroidal thickness compared to age-matched controls without Parkinson’s disease [18]. Since choriocapillaris loss predicts drusen accumulation and GA progression [19, 20], it is possible that L-DOPA treatment improves choriocapillaris perfusion through choroidal dilation. Additionally, Parkinson’s disease patients have inner retinal atrophy compared to age matched controls [18]. It is possible that inner retinal atrophy leads to greater perfusion of the outer retina from the retinal circulation, improving relative ischemia and preventing GA. Furthermore, exosomes are secreted from the apical and basolateral surfaces of the RPE and contribute to drusen development and AMD progression [21]. Since L-DOPA agonism of GPR143 reduces exosome release from the RPE [11], L-DOPA exposure may protect from GA progression via reduced exosome production. Finally, L-DOPA can reduce oxidative stress in RPE cells [16], which is another potential mechanism of L-DOPA protection against GA.

Time to GA development was 1.7 years in control eyes and 2.1 years in L-DOPA exposed eyes (Table 1). We hypothesize that since the majority of L-DOPA exposed eyes developed GA in the first 2 years (26 of 40 total eyes) that L-DOPA exposed eyes were referred at a later disease time point to a retinal specialist compared to control eyes. Unfortunately, we do know how long these eyes were exposed to L-DOPA prior to referral to a retinal specialist. Therefore, future studies are needed to investigate the timing between L-DOPA exposure and GA development.

In addition to L-DOPA exposure, we identified younger age as a protective factor and intermediate AMD as well as AREDS2 use as risk factors for new-onset GA detection. Drusen types that would be classified as intermediate rather than early AMD are associated with GA incidence and progression [22–24]. Similarly, older age is associated with new-onset GA [22, 24]. Contrary to our data, AREDS supplements do not have a significant effect on the development or progression of GA [4]. We suspect that this is a confounding result where intermediate AMD stage and the use of AREDS2 vitamins are highly correlated and their individual effects cannot be deciphered.

There were several limitations with our study. First, like all database studies, the accuracy of our results is dependent upon the quality of data available in the Vestrum Health Retina Database. Since fundus photos, optical coherence tomography, and fundus autofluorescence were not available for confirmatory review in the Vestrum deidentified database, our results are reliant on diagnosis coding. Second, patients with Parkinson's disease may not return to the eye clinic for follow-up appointments because of reduced mobility, which would reduce the detection of new-onset GA, and/or mortality. However, our use of PSM matched the control and L-DOPA exposed groups so that each group had an identical follow up period (Table 1, 2.6 years for control and 2.5 years for L-DOPA), partially negating this limitation. Third, our analysis only controlled for age, sex, smoking status, AREDS use, and AMD stage as covariates. We cannot rule out the influence of other covariates like co-existing retinal disease or systemic factors. Fourth, because many of the systemic past medical histories were incomplete, it is unknown how the stages of Parkinson's Disease were distributed; thus, the impact of Parkinson’s Disease stage on new-onset GA is unknown. Finally, since patients with Parkinson’s disease are frequently prescribed L-DOPA, we cannot rule out that Parkinson's disease and not L-DOPA medication reduces new-onset GA through improved choroidal perfusion.

## Conclusions

In summary, L-DOPA exposure was associated with a 32% reduced likelihood of progression to new-onset GA. These findings support the role of L-DOPA as a possible protective therapy against AMD progression, including neovascular AMD and GA. Future studies will additionally investigate if L-DOPA can reduce progression of early AMD to intermediate AMD.

## Data Availability

The datasets used and/or analyzed during the current study are available from the corresponding author on reasonable request.

## Abbreviations

AMD: Age-related macular degeneration
GA: Geographic atrophy
L-DOPA: Levodopa
AREDS2: Age-related eye disease study 2
HR: Hazard ratio
CI: Confidence interval
RPE: Retinal pigment epithelium
PSM: Propensity score matching
ICD: International classification of disease
VEGF: Vascular endothelial growth factor
OR: Odds ratio
